# Adopting the 4Ms Framework and Age-Friendly Concepts Into Dental Care

**DOI:** 10.1093/geroni/igab046.1600

**Published:** 2021-12-17

**Authors:** Szilvia Arany, Annette Medina-Walpole, Thomas Caprio

**Affiliations:** 1 Specialty Care, General Dentistry, Eastman Institute for Oral Health, University of Rochester, Rochester, NY, New York, United States; 2 University of Rochester, Rochester, New York, United States; 3 University of Rochester Medical Center, Rochester, New York, United States

## Abstract

We report on launching a dental initiative that addresses healthy aging and oral health, focusing on prevention and changing the false belief that aging inevitably involves deterioration in oral health. Our project integrates the age-friendly health system's principles into specialty dental care at Eastman Institute for Oral Health, URMC. We established an oral health-centered framework with project elements of the 4Ms - what matters, medication, mentation, and mobility. We developed pilot 4Ms templates integrated into dental charts to implement healthy-aging key oral health processes, including oral health assessment, treatment planning, and oral hygiene support. We engaged all members of the dental team through educational sessions for providing care for patients affected by Alzheimer’s disease and related dementias. We leveraged on locally available interdisciplinary resources and the collaboration reflecting undergoing efforts at the University of Rochester Division of Geriatrics and the Finger Lakes Geriatric Education Center, a Geriatric Workforce Enhancement.

